# Temporal Constraints of Behavioral Inhibition: Relevance of Inter-stimulus Interval in a Go-Nogo Task

**DOI:** 10.1371/journal.pone.0087232

**Published:** 2014-01-29

**Authors:** Francisco Zamorano, Pablo Billeke, José M. Hurtado, Vladimir López, Ximena Carrasco, Tomás Ossandón, Francisco Aboitiz

**Affiliations:** 1 Centro Interdisciplinario de Neurociencias, Pontificia Universidad Católica de Chile, Santiago, Chile; 2 Departamento de Psiquiatría, Escuela de Medicina, Pontificia Universidad Católica de Chile, Santiago, Chile; 3 Centro de Investigación en Complejidad Social, Facultad de Gobierno, Universidad del Desarrollo, Santiago, Chile; 4 Clínica Alemana, Universidad del Desarrollo, Santiago, Chile; 5 Escuela de Psicología, Pontificia Universidad Católica de Chile, Santiago, Chile; 6 Instituto de Sistemas Complejos de Valparaíso, Valparaíso, Chile; 7 Servicio de Neurología y Psiquiatría, Hospital Luis Calvo Mackenna, Facultad de Medicina, Universidad de Chile; Cardiff University, United Kingdom

## Abstract

The capacity to inhibit prepotent and automatic responses is crucial for proper cognitive and social development, and inhibitory impairments have been considered to be key for some neuropsychiatric conditions. One of the most used paradigms to analyze inhibitory processes is the Go-Nogo task (GNG). This task has been widely used in psychophysical and cognitive EEG studies, and more recently in paradigms using fMRI. However, a technical limitation is that the time resolution of fMRI is poorer than that of the EEG technique. In order to compensate for these temporal constraints, it has become common practice in the fMRI field to use longer inter-stimulus intervals (ISI) than those used in EEG protocols. Despite the noticeable temporal differences between these two techniques, it is currently assumed that both approaches assess similar inhibitory processes. We performed an EEG study using a GNG task with both short ISI (fast-condition, FC, as in EEG protocols) and long ISI (slow-condition, SC, as in fMRI protocols). We found that in the FC there was a stronger Nogo-N2 effect than in the SC. Moreover, in the FC, but not in the SC, the number of preceding Go trials correlated positively with the Nogo-P3 amplitude and with the Go trial reaction time; and negatively with commission errors. In addition, we found significant topographical differences for the Go-P3 elicited in FC and SC, which is interpreted in terms of different neurotransmitter dynamics. Taken together, our results provide evidence that frequency of stimulus presentation in the GNG task strongly modulates the behavioral response and the evoked EEG activity. Therefore, it is likely that short-ISI EEG protocols and long-ISI fMRI protocols do not assess equivalent inhibitory processes.

## Introduction

The ability to respond rapidly based on little information is essential in daily life and activities, like retracting our bodies after a loud noise in order to protect ourselves, or anticipating an opponent's move in sports. However, in some situations the inhibition of our instinctive reactions or automatic responses is mandatory to achieve a specific goal. In general, these processes belong to a cluster of behaviors called behavioral inhibition (BI), which is defined as comprising three interrelated processes: (i) inhibiting the initial prepotent response to an event, so-called response inhibition (RI); (ii) stopping an ongoing response, which thereby permits a delay in the decision to respond; and (iii) self-directed responses that result from competing events and responses or interference control (IC) [1], [2]. One of the most used cognitive paradigms to explore inhibitory processes involved in BI is the Go-Nogo task (GNG) [3], [4]. This task has been paramount in the clinical characterization of psychiatric disorders like ADHD [5], and in unraveling their underlying neural mechanisms, because it provides information about motor preparedness and response, inhibition, and executive mechanisms [6]. Furthermore, the GNG task has been conducted under different experimental manipulations in order to characterize the cognitive processes underlying response inhibition [7]. These studies have focused mainly on the effects of cueing [8]–[10], trial sequence effect and expectation [11], [12], Go/Nogo trial probabilities [13]–[15], salience of stimuli [16], [17], perceptual similarity of stimuli [18]–[20], and stimulus and response modalities [19]–[24]. However, despite the amount of evidence generated, debate is still open about the automatic or controlled nature of response inhibition [4] and about the contribution of motor and attentional systems to RI [7].

The GNG task, originally a tool in psychophysics, has been successfully applied in conjunction with brain mapping tools such as EEG and fMRI. In pure psychophysics, the task focuses in the behavioral differences of performance and reaction time (RT), while its use in conjunction with EEG techniques has allowed the study of event-related potential (ERP) components associated with inhibition, attention and cognitive processing like N2 and P3. In contrast to the rapid sampling permitted by EEG, fMRI techniques are notorious for their low temporal resolution. The slow kinetics of the hemodynamic response associated with neural activity [25], together with the limited acquisition rate for whole brain scans require a lower rate of stimuli presentation in order to detect effects using fMRI. This temporal constraint in fMRI has led to important modifications in the interstimuli-timing schedule for GNG experiments from that used in classical behavioral/EEG paradigms. Whereas the classical paradigm uses ISIs of hundreds of milliseconds [21], [26], most of the fMRI studies increase the ISI to several seconds [3], [11], [27]–[29].

The dramatic differences in the ISIs used in fMRI and EEG approaches of the GNG task raise the question of whether there are qualitative or quantitative differences in the cognitive mechanisms underlying behavioral inhibition when using either technique. In one scenario both techniques may be probing the same cognitive mechanism only with different degrees of temporal stress. In an alternative scenario, different mechanisms are engaged as a function of temporal delay. The aim of this study is to scrutinize how variation in the temporal parameters (e.g. ISI) used in EEG and fMRI studies modulates the nature of the inhibitory process measured.

## Materials and Methods

### Participants

Twenty normal adult subjects, all right-handed Spanish speakers, 6 female, 14 male, ages 18–31 years (mean 24.6), participated in the study. All participants had normal or corrected-to-normal vision, no color-vision deficiency, no history of neurological diseases, and no current psychiatric diagnosis or psychotropic prescriptions.

### Ethics Statement

Written consent was signed after detailed explanation of the scope of the study, in accordance with guidelines and procedures approved by the Ethics Committee of the Pontificia Universidad Católica de Chile. All experiments were performed at the Laboratorio de Neurociencia Cognitiva of the Department of Psychiatry of the University.

### Paradigm

**Figure 1:**
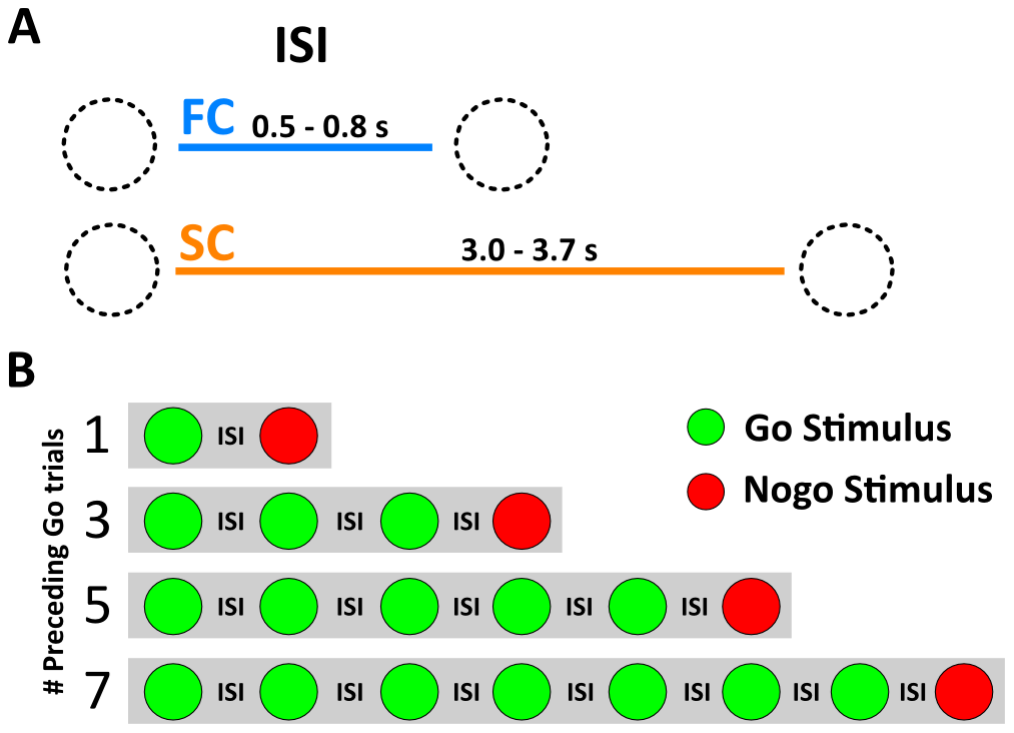
Schematic representation of the task A. Representation of ISI differences between FC and SC. B.- Diagram of sequences used for stimulus presentation.

Participants performed two versions of the Go-Nogo task, a Fast Condition (FC) and a Slow Condition (SC) that differed only in their inter-stimuli interval (ISI). ISIs were drawn from uniform distributions, 500–800 ms interval for the FC and 3000–3700 ms interval for the SC (Figure 1A). Stimulus duration was 300 ms indistinctly for Go and Nogo trials in both FC and SC. Each participant performed four randomly alternated blocks of 300 trials, two FC and two SC, each one.

Our experimental paradigm consisted of a serial presentation of screen centered green or red colored circles (3.5° of visual arc) for Go and Nogo stimuli, respectively. Nogo trials were preceded by sequences of 1, 3, 5 Go trials as indicated in Durston and colleagues [11]; in addition we used a fourth condition with 7 Go trials (Figure 1B).

Stimuli were presented in a 21″ CRT monitor positioned 57 cm in front of the subject. The subjects were instructed to press a button as fast as possible after a Go stimulus (green circle) and to restrain after a Nogo stimulus (red circle). The same instruction was provided in both FC and SC. Two consecutive Nogo stimuli were never presented. The tasks were programmed in Presentation 13.0 (Neurobehavioral Systems, Inc.).

### Electrophysiological Recordings

Continuous EEG recordings were obtained with a 40-electrode NuAmps EEG System (Compumedics Neuroscan). All impedances were kept below 5 kΩ. Electrode impedance was retested during pauses to ensure stable values throughout the experiment. All electrodes were referenced to averaged mastoids during acquisition and the signal was digitized at 1 kHz. Electro-oculograms (EOG) were obtained from four electrodes arranged in two bipolar derivations, HEOG and VEOG [30]. All recordings were obtained using Scan 4.3 (Compumedics Neuroscan) and stored for off-line treatment. Later, EEG signals were preprocessed using a 0.1–100 Hz band-pass filter. Eye blinks were identified by a threshold criterion of ± 100 µV, and their contribution was removed from each dataset using principal components analysis by singular value decomposition and spatial filter transform. Other remaining artifacts (e.g., muscular artifacts) were detected by visual inspection of the signal and the trials that contained them were removed. Thus, we obtained 220±20 artifact-free trials per subject for FC and SC.

### Behavioral Statistical Analysis

All behavioral statistical analyses were performed in R software (http://www.R-project.org). For a better characterization of the Go trial RTs measured in FC and SC, we perform an ex-Gaussian analysis as follows. First, a smoothed RT density distribution was obtained by convolving the raw RT distribution per subject and condition with a Gaussian kernel (standard deviation of 10 ms) in steps of 1 ms from 100 to 600 ms. Then, we computed a Wilcoxon signed rank test between conditions across subjects for each temporal bin, and corrected the resulting p-values using the Bonferroni multiple comparison criterion. Additionally, we fitted an ex-Gaussian distribution per subject and condition. This distribution is the sum of a normal and an exponential distribution, and is defined by three parameters: Mu, Sigma (mean and standard deviation of the normal part of the distribution, respectively) and Nu (decay constant of the exponential part of the distribution) [31], [32]. We finally compared these parameters between conditions using a Wilcoxon signed rank test.

**Figure 2:**
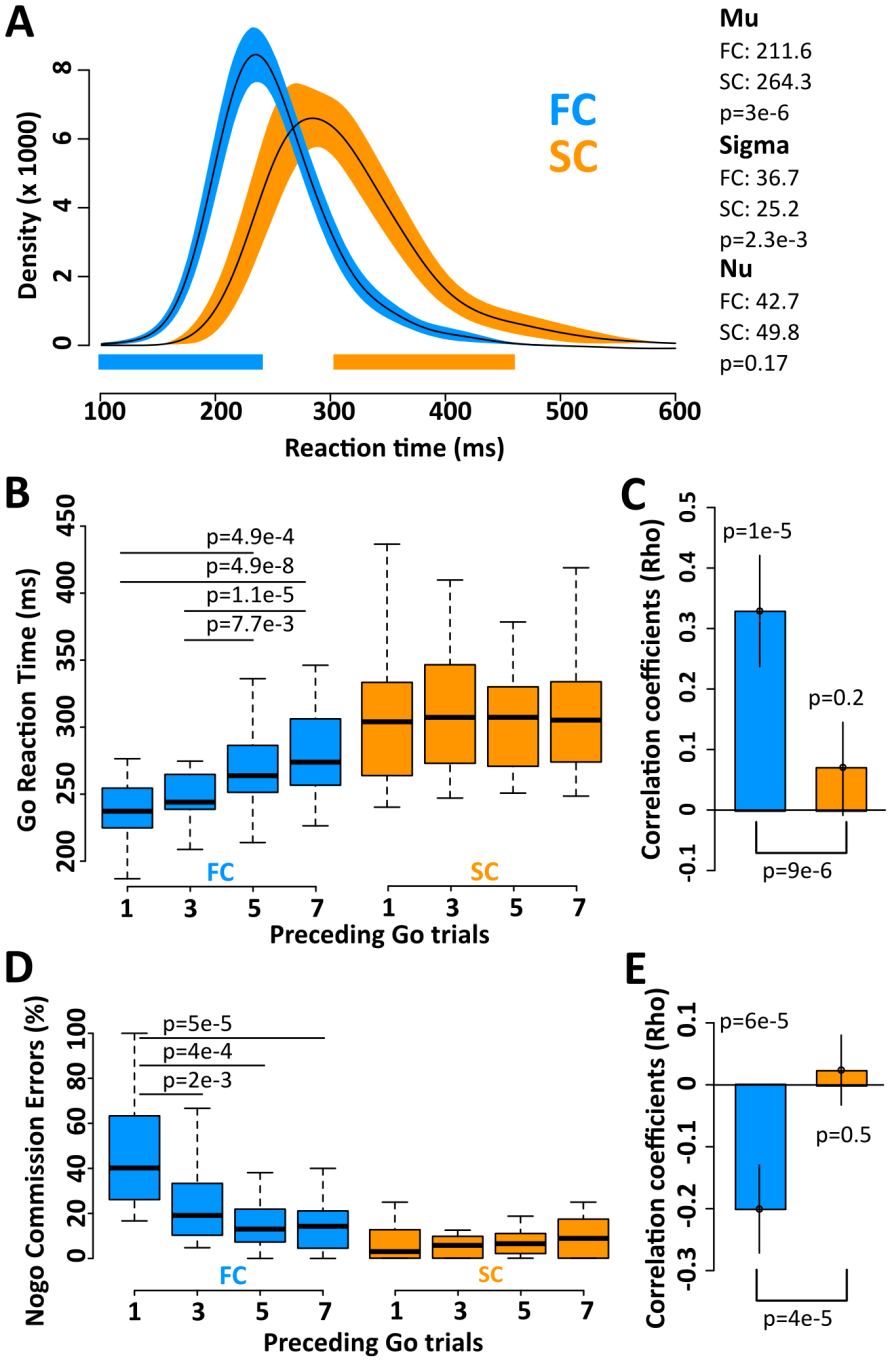
Behavioral results. A. Density distribution for reaction times to Go trials in FC (blue), and SC (orange). The bar below represents regions where the distributions are significantly different (blue, FC > SC; orange, SC >FC; p<0.05, Bonferroni corrected). B. Go trial reaction times classified by the number of consecutive Go trials that preceded a Nogo trial (1,3,5 and 7) for FC (blue) and SC (orange). C. Correlation coefficients (Rho) between Go trials that preceded a Nogo trial and the respective Go trial reaction time, calculated per each subject and condition. D. Nogo trial errors classified by the number of consecutive Go trials that preceded a Nogo trial (1,3,5 and 7) for FC (blue) and SC (orange). E. Correlation coefficients (Rho) between Nogo trial errors and number of Go trials that preceded a Nogo trial, calculated per each subject and condition. B, D. Boxes represent the interquartile range and the whiskers the most extreme data points. C, E. Error bar represent the 95% confidence interval by bootstrapping distributions.

For behavioral data analysis, Go trial RT and Nogo trial commission errors (CE) were grouped according to the number of Go trials that preceded the Nogo trial in each sequence. For RT analysis we used only Go trials that preceded Nogo trials (i.e. the last Go trial of the sequence) in order to obtain a similar number of trials per condition. We used both (i) the mean value per subject, comparing them by the Friedman rank sum test, and (ii) the single trial values computing a Spearman correlation per subject and condition. Finally, to compare the Rho values obtained we performed a Wilcoxon signed rank test. All tests were two-tailed. In bar plot of Rho values (Figures 2C, E), we show the 95% confidences intervals of the means obtained by bootstrapping distribution.

### EEG Data Analysis

All algorithms were implemented in MATLAB 7.0. (MATLAB, version 7.0 (r14) ed. Natick, Massachusetts: The Mathworks Inc., 2004), by in-house scripts used in our previous work [33] (available online http://lantoolbox.wikispaces.com/). Evoked activity was computed as the mean of the electrical brain activity for each electrode and each participant over trials.

To determinate differences between the ERPs elicited by Go and Nogo trials in FC and SC, we conducted paired comparisons (Wilcoxon signed-rank tests, two-tailed) across the mean per condition per subject at each temporal bin. Then, we corrected for multiple comparisons using a cluster based permutation test [34]. In this method, the data was transformed to a three-dimensional space (two-dimensional plane of electrode positions and time) and clusters of continuous significant areas were defined by pooling neighboring bins showing the same effect (p<0.05). The cluster-level statistics was the sum of the individual statistics of all bins within the corresponding cluster. We controlled for the false alarm rate of the complete spatio-temporal cluster (set of electrodes and time bins) by evaluating the cluster-level significance under the permutation distribution of the cluster that had the largest cluster-level statistics. The permutation distribution was obtained by randomly permuting all trials of the two conditions for each subject thereby eliminating any systematic difference between the conditions. After each permutation, a Wilcoxon signed-rank test was computed across the mean per permuted-conditions per subject. After 1000 permutations, the cluster-level significance was estimated as the proportion of elements of the permutation distribution greater than the observed cluster-level significance.

The N2 amplitude was calculated using the major negativity of the FCz electrode between 200 and 300 ms after stimulus presentation. In the same way the P3 amplitude was calculated using the major positivity of the FCz electrode between 300 and 400 ms after stimulus presentation, for every subject and condition. In the analysis of Nogo-P3 amplitude classified by the number of preceding Go trials we used the Friedman rank sum test, and the Spearman correlation test per subject and condition (using the number of preceding Go trials as ordinal variable). Finally, we used the Wilcoxon signed rank test for Rho values comparison. To study the relationship between Go-N2, Go-P3, Nogo-N2, Nogo-P3 amplitudes, number of preceding Go trials, Go trial RT and the Nogo trial CE, we used the Spearman partial correlation test among all variables for both FC and SC. To compare the partial correlation obtained from FC and SC, we used a permutation test as follows. First, we calculated the strength of all links in both conditions by taking the sum of the squared Rho values of every correlation. Second, we contrasted the observed absolute difference between conditions with a distribution obtained by randomly permuting the two conditions (FC and SC) by subject. Finally, we calculated the p-values as the proportion of the absolute differences in the permutation distribution greater than or equal to the observed difference between conditions. Thus, we were able to control that the observed differences in the link strength between conditions were not due to chance.

## Results

### Behavioral study

To assess whether the change of ISI impacts the behavior of subjects, we first studied the accuracy (rate of successful trials) across subjects for different conditions. We did not find differences between FC and SC in Go trials (p = 0.25, Wilcoxon signed rank test); however, in Nogo trials we observed a significant decrease in accuracy in the FC compared with the SC (p = 1.37e-2). We also found that reaction times were significantly shorter in FC Go trials than in SC Go trials. To examine this difference in detail we performed an ex-Gaussian analysis of reaction times in the FC and SC (Figure 2A). This analysis showed significant differences in Mu (p = 3e−6) and Sigma (p = 2e−3) parameters, which represent the mean and standard deviation of the Gaussian part of the distribution respectively. The Nu parameter, that represents the time decay of the exponential part of the distribution, showed no significant difference, indicating that both conditions exhibit a similar tendency to obtain long reaction times.

In the FC, the number of Go trials before a Nogo stimulus modulated both the Go trial RT and the Nogo trial CE. Thus, in FC sequences, the first responses were significantly faster (p = 3.3e−9, Friedman rank test) but more inaccurate than the latter (p = 7.4e−6). None of these effects were observed in the SC (p = 0.11 for RT and p = 0.25 for CE) (Figure 2B, 2D). To assess the strength of the effects, we computed the Spearman rank correlation coefficient (Rho) between the number of preceding Go trials and both Go trial RTs and Nogo trial CEs, for each subject and each condition. In accordance with the previous results, only in the FC, the Rho values of the correlation between the number of preceding Go trials and their RT differed significantly from zero (p = 1e−5), yielding a significant difference between these Rho values for FC and SC (p = 9.0e−6) (Figure 2C). In the same way, for CE in Nogo trials, only in the FC the Rho values were significantly different from zero (p = 6e−5), and the Rho values for FC and SC were significantly different (p = 4e−5, Figure 2E). Finally, we evaluated the correlation between Go trial RT and Nogo trial CE. In this analysis, the Rho coefficients were significantly different between conditions (mean Rho for FC = −0.23; mean Rho for SC = −0.09; p = 0.01, Wilcoxon signed rank test).

Summarizing these results, (i) in the FC, there were shorter overall reaction times to Go trials and more commission errors to Nogo trials than in the SC; (ii) only in the FC, Go trials RT and Nogo CE were significantly correlated with the number of preceding Go trials.

### Electroencephalographic study

**Figure 3:**
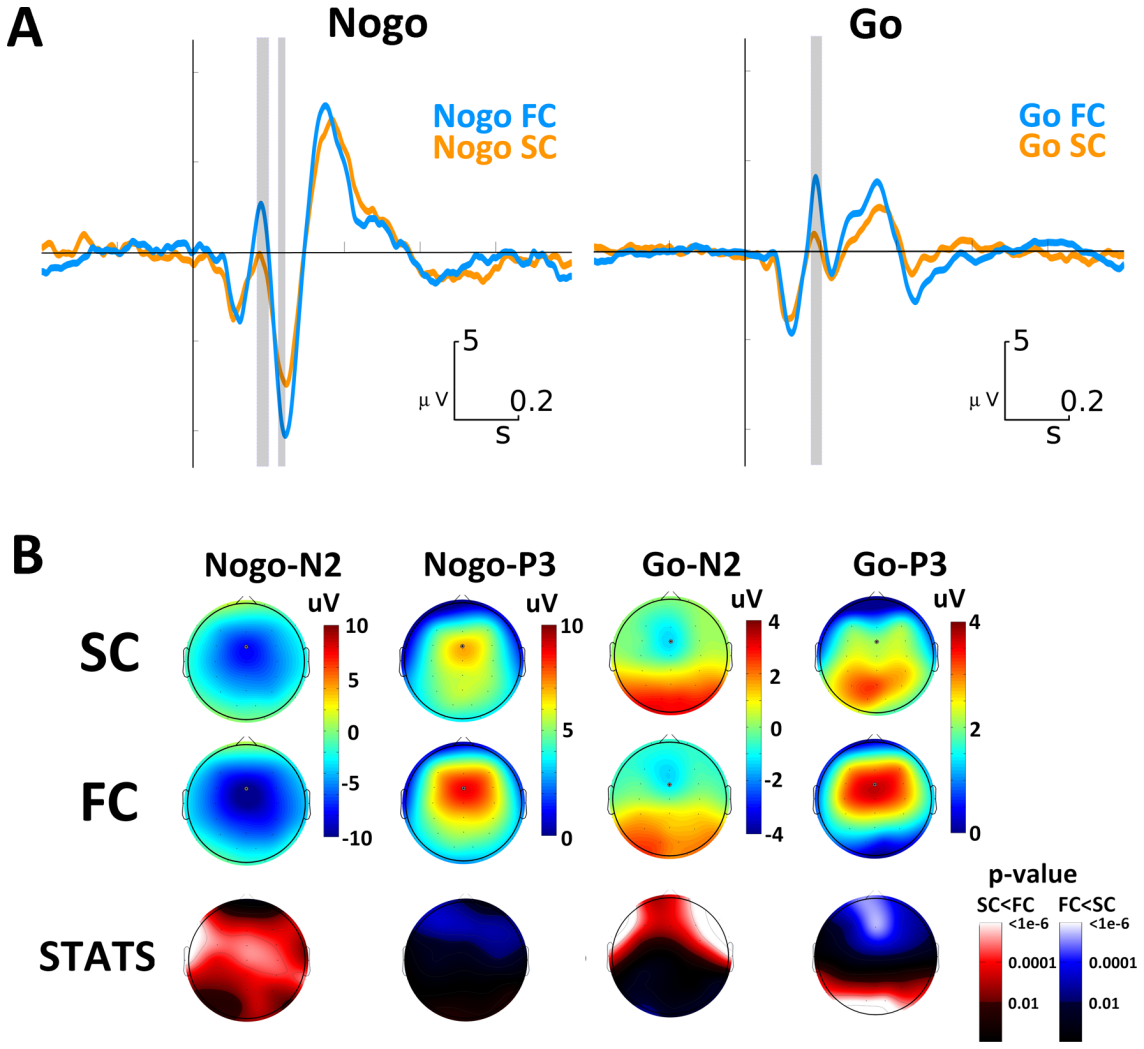
ERP results for all Go and Nogo trials. A. ERP for Nogo and Go trials in FC (blue line) and SC (orange line), measured at FCz electrode. B. Topographic distributions of Nogo-N2, Nogo-P3, Go-N2 and Go-P3 for FC (first raw) and SC (second raw). Statistical differences between FC and SC topographies are depicted in the third row.

The ERPs for Go and Nogo stimuli in both FC and SC are illustrated in Figure 3A. Figure 3B displays the scalp topographies of N2 and P3 for all conditions. In line with previous studies, overall higher amplitudes were evidenced for Nogo than for Go ERP late components (N2, P3)[21].

Additionally, when comparing FC and SC in both Go and Nogo conditions, early components (P1, N1) showed statistical differences. Whereas, both Go and Nogo stimuli elicited a P1 ERP component whose amplitude was significantly greater in FC than in SC, the amplitude of N1 ERP component was significantly greater in SC than in FC (Figure S1).

On the other hand, when comparing late inhibition-related components elicited by both Go and Nogo stimuli, only Nogo-N2 was significantly greater in FC (see Figure 3A). Neither N2 nor P3 evidenced statistical differences between FC and SC in the Go condition.

**Figure 4:**
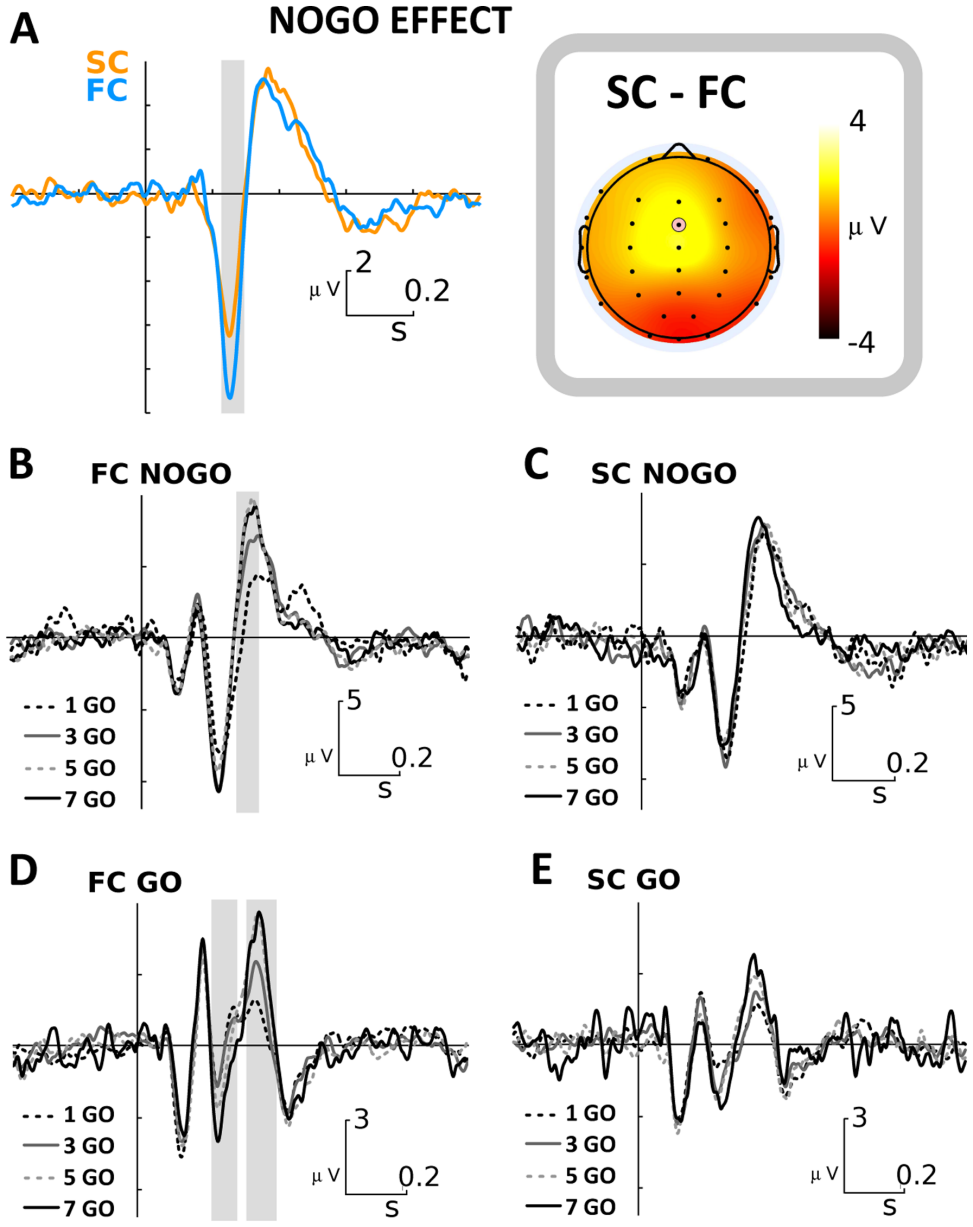
ERP results for Go and Nogo trials classified by preceding Go trials. A. ERP difference between Nogo and Go trials (Nogo Effect) in FC (blue line) and SC (orange line), measured at FCz electrode. B, C. ERPs elicited by Nogo stimuli when 1,3,5, or 7 Go trials preceded a Nogo trial, in the FC (B) and in the SC (C), measured at FCz electrode. D, E. ERPs elicited by the 1st, 3th, 5th, or 7th consecutive Go stimuli, in the FC (D) and in the SC (E). A–D. The gray bar indicates regions of statistical significance (p<0.05, cluster based permutation test).

In order to characterize the inhibitory processes involved in the Go-Nogo task for FC and SC, we studied the Nogo-effect, which contrasts Nogo and Go activities. In this way the inhibitory activity reflected in N2 and P3 components emerges clearly for both FC and SC conditions (Figure 4A). We found that the Nogo-N2 was significantly larger in FC than in SC (p<0.01 cluster based permutation test for multiple comparisons correction). The difference for the Nogo effect between FC and SC is depicted in the right panel of Figure 4A. No statistically significant differences were observed for the Nogo-P3 component in a preliminary analysis that included all Nogo trials for FC and SC. Considering this, we additionally performed a specific analysis taking into account the possible increased expectative after each Go trial and the increased effort to inhibit interferences.

Thus, we studied the Nogo-P3 amplitude when preceded by 1, 3, 5, and 7 Go stimuli for both FC and SC, and observed a parametric increase of the Nogo-P3 amplitude only in the FC (Figure 4B). This amplitude modulation effect did not occur in the SC (Figure 4C). In a similar way, we studied the ERPs elicited by Go trials, with the difference that in this case we studied the ERPs generated by the first, third, fifth and seventh consecutive Go stimuli of each sequence. As in the previous case, the Go-P3 amplitude was modulated by the length of Go-trial sequences only in the FC. Remarkably, we also found a parametric increase of the Go-N2 amplitudes in the same condition (Figure 4D, E).

**Figure 5:**
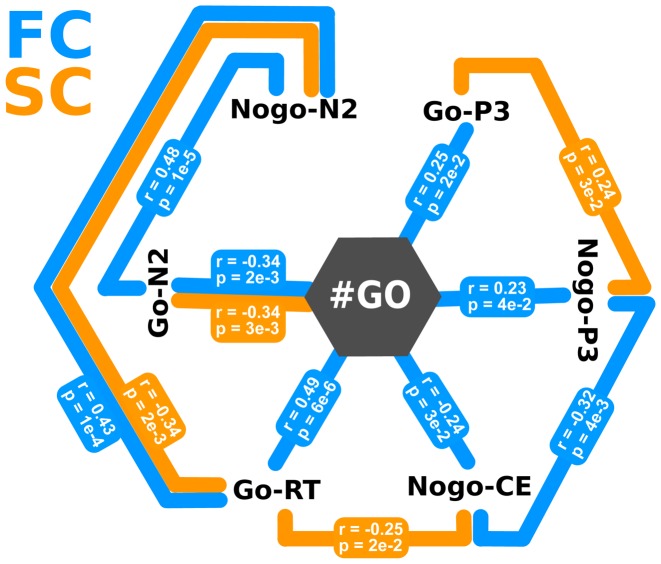
Spearman partial correlation analysis for FC and SC. Solid color lines indicate significant correlation coefficients between variables (blue for FC and orange for SC).

Finally, in order to obtain a better characterization of the relation among independent (number of preceding Go trials) and dependent variables (Go-N2, Go-P3, Nogo-N2, Nogo-P3 amplitude, Go trial RT and Nogo CE) in both FC and SC, we performed a Spearman partial correlation analysis (Figure 5). On one hand, in the FC we found that the number of preceding Go trials correlated significantly and positively with Go-P3 (p = 2e−2; r = 0.25) and Nogo-P3 amplitude (p = 4e−2; r = 0.23) and RT (p = 6e−6; r = 0.49), and negatively with Go-N200 (p = 2e−3; r = −0.34) with the CE (p = 3e−2; r = −0.24). The amplitude of the Nogo-N2 component showed a significant correlation only with the previous Go-RT (p = 1e−4; r = 0.43) and Go-N2 amplitude (p = 1e−5; r = 0.48). Additionally, the CE also shows a negative and significant correlation with the amplitude of the Nogo-P3 component (p = 4e−3; r = −0.32). On the other hand, most of the significant correlations obtained in the FC were not replicated in SC. Nonetheless, the Go-RT were inversely correlated the Nogo-N2 amplitude (p = 2e−3; r = −0.34) and the Go-N2 amplitudes were also modulated by the number of preceding Go trials (p = 3e−3; r = 0.34); additionally RT was significantly and negatively correlated with CE (p = 2e−2; r = −0.25), and the amplitudes of Go-P3 and Nogo-P3 were significantly correlated (p = 3e−2; r = 0.24). To verify that the differences in the partial correlation analysis between FC and SC were not due to chance, we performed a permutation test (see methods). This analysis indicated that the differences between the strength of overall links in FC and SC partial correlations were significant (p = 3e−2). In other words, the variables were more strongly correlated among them in FC than in SC.

Summarizing these findings, (i) there was a larger N2 response to Nogo trials in the FC than in the SC, but no amplitude modulation by the number of preceding Go trials was observed in any of the two conditions; (ii) amplitude modulation was observed for Go-N2 in both FC and SC, and for Go-P3 and Nogo-P3 amplitudes in FC but not in SC; (iii) only in the FC, all dependent variables measured (Go-N2, Go-P3, Nogo-P3, Go trial RT and Nogo CE), with the exception of the Nogo-N2 amplitude, correlated significantly with the number of preceding Go trials (Figure 5).

## Discussion

The main objective of this article has been to examine whether variations of the inter-stimulus interval modify the nature of the inhibitory process that operates during the Go–Nogo task. Originally, the Go–Nogo task was purported to measure response inhibition, which is only one component of behavioral inhibition [35]. It was not intended to assess other components, such as inhibiting interferences or the ability to inhibit a response over a protracted period of time [1]. In the same vein, Simmonds and colleagues [6] manifested that the GNG task has been used for several decades as a measure of response inhibition in fMRI designs, although it is not clear that the activations measured contrasting Go and Nogo stimuli precisely correspond to a unique inhibition process like response inhibition. Mostofsky and colleagues [36] highlighted this issue, stressing that the GNG task especially fits the study of motor response inhibition because it minimizes cognitive demands (e.g. Working Memory) [36]. They briefly discussed the implications of using longer ISI in fMRI studies of the Go-Nogo task. However to our knowledge, this issue has never before been addressed experimentally using one single task.

Our behavioral results reveal a major effect of the ISI on the performance dynamics as expressed by Go trial RT and Nogo trial CE. In concordance with previous works that demonstrate that subjects respond slowly in Go trials when conflict is present [37]–[39], we observe a parametric increment of the RT in dependence of the Go trials elapsed only for the FC of the GNG task (see Figure 2).

In the FC, the larger the number of Go trials preceding a Nogo stimulus, the longer the Go trials RT and fewer the Nogo trials CE. By contrast in the SC, Go trial RT and Nogo trial CE were independent of the number of preceding Go trials. These findings suggest that the low stimuli presentation frequencies, typical of fMRI/Go-Nogo studies, undermine the emergence of response automation, reducing the need to inhibit the prepotent motor act, and support the idea that in Go-Nogo studies with lower prepotency, successful stopping may be more about “deciding” not to go than countermanding and initiated response [39].

In addition, previous studies have shown that subject performance decreases with longer ISIs [40]. Then, a longer ISI could also favor the occurrence of attentional interference processes, forcing the subject to suppress irrelevant endogenous and exogenous stimuli in order to fulfill the test. Nevertheless, whether the concept of inhibition is relevant in the suppression of activated cognitive contents or in the control of interfering information is still being disputed [7]. In line with this interpretation is the evidence from the cognitive-energetic model for ADHD [41], which posits that event rate alters the energetic state of the subject promoting fast-inaccurate responding at high rates, and slow-inaccurate responding at low rate event presentation.

Previous ERP studies indicated that behavioral inhibition is associated to the N2 component, whose amplitude increases in Nogo trials according to the response inhibition requirements [26], [42]–[44], as compared to Go trials where no RI activity is needed [15].

In particular, the Nogo-N2 has been interpreted to represent motor inhibition [45]–[47], detection of response conflict [15], [45], [48], processing a mismatch between a stimulus an a mental template [49] or cognitive resources allocation [50]. On the other hand, Go-N2 may represent the strength of motor preparation processes, which seems to vary as a positive function of the probability of the Go stimulus [9]. For a comprehensive review about existent literature on response inhibition tasks and EEG studies see Huster and colleagues [7].

In the current study the amplitude of the N2 Nogo-effect was significantly different when comparing FC and SC (Figure 4A). The larger N2 effect in FC could be explained by the greater effort required to withhold the Go response [51]. Additionally, when we studied in detail the Nogo-N2 amplitude elicited by 1, 3, 5, and 7 preceding Go stimuli, we observed significant modulations only in the FC (Figure 4B, 4C), and this was confirmed by the partial correlation analysis (Figure 5).

Our results are in line with those reported in previous works [52] suggesting that N2 may represent attentional rather than cognitive control processing [49]. We observed an absence of Nogo-N2 amplitude modulation by the number of preceding Go trials for both FC and SC, but there was an overall higher amplitude in FC. This effect could also be interpreted as resulting from a higher level of arousal in the latter condition, revealing the susceptibility of this component to the attentional state but not necessarily to the cognitive interference control processing. A similar amplitude modulation was found in the earlier P1 and N1 components (Figure S1). That said, it is noticeable that a negative and significant correlation was found in both the FC and SC between Go-N2 amplitude (but not for Nogo-N2) and the number of preceding Go trials. These might be reflecting local changes in the expectancy or probability of occurrence of a Nogo trial, and the resulting modification of attentional resources allocation [7]. The Stimulus-driven N2 amplitude modulation has been interpreted as part of adjustments related to what is called the “Stimulus Set” and differs from those related with the “Response Set”. This interpretation concurs with earlier discussions on the significance of N2 and P3 and the differential modulation of these two components [53]. While perceptual load and conflict monitoring might broadly modulate N2, the P3 seems more dependent on the cognitive load and response preparation [7], [53]. Furthermore, increased frontal-midline N2 has been observed whenever an event is unexpected, irrespective for whether it is a Go or Nogo stimulus. In contrast, in these designs, the P3 effect does not seem to be affected in the same way [12]. Other authors have suggested that the amplitude of the Nogo-N2 and Nogo-P3 vary as a negative function of Nogo stimulus probability [15], while the Go P3 varies as a negative function of Go stimulus probability [9].

One of the first phenomenological descriptions proposed that P3 amplitude represented cognitive load and cognitive effort [54], and since then it has been traditionally associated with updating working memory [55], [56], attentional processes [44], [57], [58], and inhibitory processes [7], [10], [45], [59]–[63]. Based on current evidence two things can be said, with relative certainty, about P3: (i) P3 does not represent a single cognitive process and, (ii) its interpretation will vary depending on experimental and task settings.

Since withholding responses after a Nogo stimulus presentation is a common task requirement, it is not surprising that a Nogo-P3a component was elicited in both FC and SC. Furthermore, the amplitude difference observed between FC and SC can be explained in terms of the strength of response inhibition [14], and as a reflect of the amount of allocated cognitive resources [50]. In contrast to the Nogo stimuli, we found a strong divergence between FC and SC Go-P3 topography. Our results showed that the maximum amplitude of the Go-P3 component in FC was found over the mid-frontocentral region of the scalp, whereas that Go-P3 in SC showed a mid-centroparietal distribution (Figure 3B). These two P3 scalp topographies have been extensively described before as P3 subcomponents named P3a and P3b respectively. While P3a may reflect a stimulus-driven disruption of frontal attention engagement mediated by dopaminergic activity, P3b should reflect both attentional and memory processing, related to the norepinephrinergic system (for a review see Polich 2007).

In our view, the “posteriorization” of Go-P3 observed in SC (Figure 3) is a reflex of a different processing mechanisms than in FC. This topographic shift could be given by an increase of top-down processing related to overriding interfering information, or alternatively, it could reflect an increase in the expectation of the Nogo stimulus appearance in the next presentation.

Normally during experiments subjects might think about things apart from the task they are performing, especially when the task has low incentive value [64], [65]. In FC the short ISI forces the attentional focus to keep engaged in the task for both Go and Nogo stimuli, while in SC, a long ISI could facilitate the appearance of task-unrelated thoughts which are associated to high tonic NE activity [65]. Thus, the neural processing associated to Go and Nogo stimuli under two different ISI may differ.

We hypothesize that in both FC and SC, Nogo stimulus presentation captures attentional resources guided by perceptual differences or novelty, triggering phasic dopamine (evidenced by the P3a) and norepinephrine signaling. In this regard, Aston-Jones and Cohen (2005) postulated that the LC phasic response plays a role in facilitating task-relevant behavioral responses [66] (i.e. Nogo stimuli detection). Nonetheless in the FC, given the short ISI, an overactivated state could be naturally triggered as the result of the synergic participation of phasic DA and NE responses. These may be behaviorally associated with a higher level of arousal, shorter RTs and, by a tendency to false alarms when Nogo stimuli are less probable. Then, to prevent errors in the short ISI condition, the overactive response state (in FC but not in SC) should be dampened through effort control [40], [67], herein referred as cognitive control.

Similarly, processing of Go-P3 stimulus in FC and SC could imply two different neurotransmitter dynamics. In a first instance, incoming stimuli invoke top-down attention switching, and bottom-up memory-driven operations that might guide response organization and production [63] in both FC and SC. Nevertheless, in a second instance because of the longer ISI of SC, fast transient bursts of NE necessary to maintain focused attention could derivate into increased levels of tonic NE, which has been related to the posteriorization of the P3 component [63], [68] and with decreased arousal and task performance [66]. Thus, both the posteriorization effect observed in Go-P3 (Figure 3B), and the significant increase of RT and its variability (Figure 2A) in the SC condition, may be interpreted as reflecting the involvement of tonic NE activity.

Processes that demand cognitive control are often distinguished from automatic ones, as they require the suppression of interfering or distracting stimuli that are not usually present in automatic tasks [69], [70]. Our results show that variations of the inter-stimulus interval in the Go-Nogo tasks, result in shifting from automatic processes in short ISI to interference control in long ISI conditions. Short ISI conditions require mainly automatic/attentional processes, which allow responding quickly but inaccurately. On the other hand, long ISI conditions activate different levels of cognitive control, as there is access to contextual information due to fewer temporal constraints. In addition in SC there is less need of motor inhibition (characteristic of FC) and decision-making processes could emerge naturally at the expenses of slower reaction times, improving accuracy. Thus, in contrast to the SC, the temporal constraints of the FC force to a fine-tune regulation between these two opposite processes in order to achieve an optimal performance. Therefore, RI could be functionally understood as a conflict of two opposite brain strategies oriented to solve problems in a different way. In this context, the rhythm of the task (e.g. the frequency of the stimulus presentation) is relevant to the processes involved in RI.

Thereby, our data indicates that a significant and relevant part of the response inhibition process may not be appropriately captured using long ISI as in fMRI Go-Nogo task designs. Therefore, both experimental design and results interpretation should consider the timing of the different cognitive processes, and how the technical constraints can allow us to measure faster or slower responses. A well-documented example about this issue, also using Go-Nogo tasks, corresponds to the simultaneous recording of skin conductance responses (SCR) and EEG studies [71], [72]. In this case, even when SCR and EEG can be assessed with high temporal resolution, the differences in the specific time courses and recovery times are considered an impediment for co-registration of slow autonomic emotional responses at skin level and faster cognitive activity related to response inhibition.

## Conclusion

The present work emphasizes the relevance of ISI in the nature of the inhibition processes measured with the Go-Nogo task, which has been only tangentially considered before [6], [73]. Our data indicates that due to the nature of the original design of the Go-Nogo task and the temporal constraints of the hemodynamic response, the long ISI (fMRI-like) GNG task puts a stronger load on cognitive inhibition processes, while short ISI (EEG-like) GNG tasks probe more directly response inhibition. These results highlight the need of further studies oriented to understand how fading between automatic and cognitive control processing operates in order to optimize behavior under different environmental demands.

## Supporting Information

Figure S1 **Visual ERP components.** A. ERP elicited by Go trials in FC (blue line) and SC (orange line). B. ERP elicited by Nogo trials in FC (blue line) and SC (orange line). A, B. ROI is depicted in the superior right corner of each figure. The gray bar indicates regions of statistical significance (p<0.05, cluster based permutation test). The lower panel shows the P1 and N1 topographical representations for both FC and SC, and its statistical differences.
(TIF)
