# Correction: Temporal Constraints of Behavioral Inhibition: Relevance of Inter-stimulus Interval in a Go-Nogo Task

**DOI:** 10.1371/journal.pone.0091266

**Published:** 2014-02-27

**Authors:** The PLOS ONE Staff

The Funding statement is incorrect. The publisher apologizes for the error. Please view the correct funding information below:

"This work was supported by CONICYT "Proyecto Anillo en Complejidad Social" SOC1101 and by the Millenium Center for the Neuroscience of Memory, Chile (NC10-001-F), which is developed with funds from the Innovation for Competitivity from the Ministry for Economics, Fomentation and Tourism, Chile. The funders had no role in study design, data collection and analysis, decision to publish, or preparation of the manuscript."
