# Supplementary figures and images for: Temporal Constraints of Behavioral Inhibition: Relevance of Inter-stimulus Interval in a Go-Nogo Task
Source: PLoS One. 2014 Jan 29;9(1):e87232. doi: 10.1371/journal.pone.0087232 (PMC3906165; doi:10.1371/journal.pone.0087232)

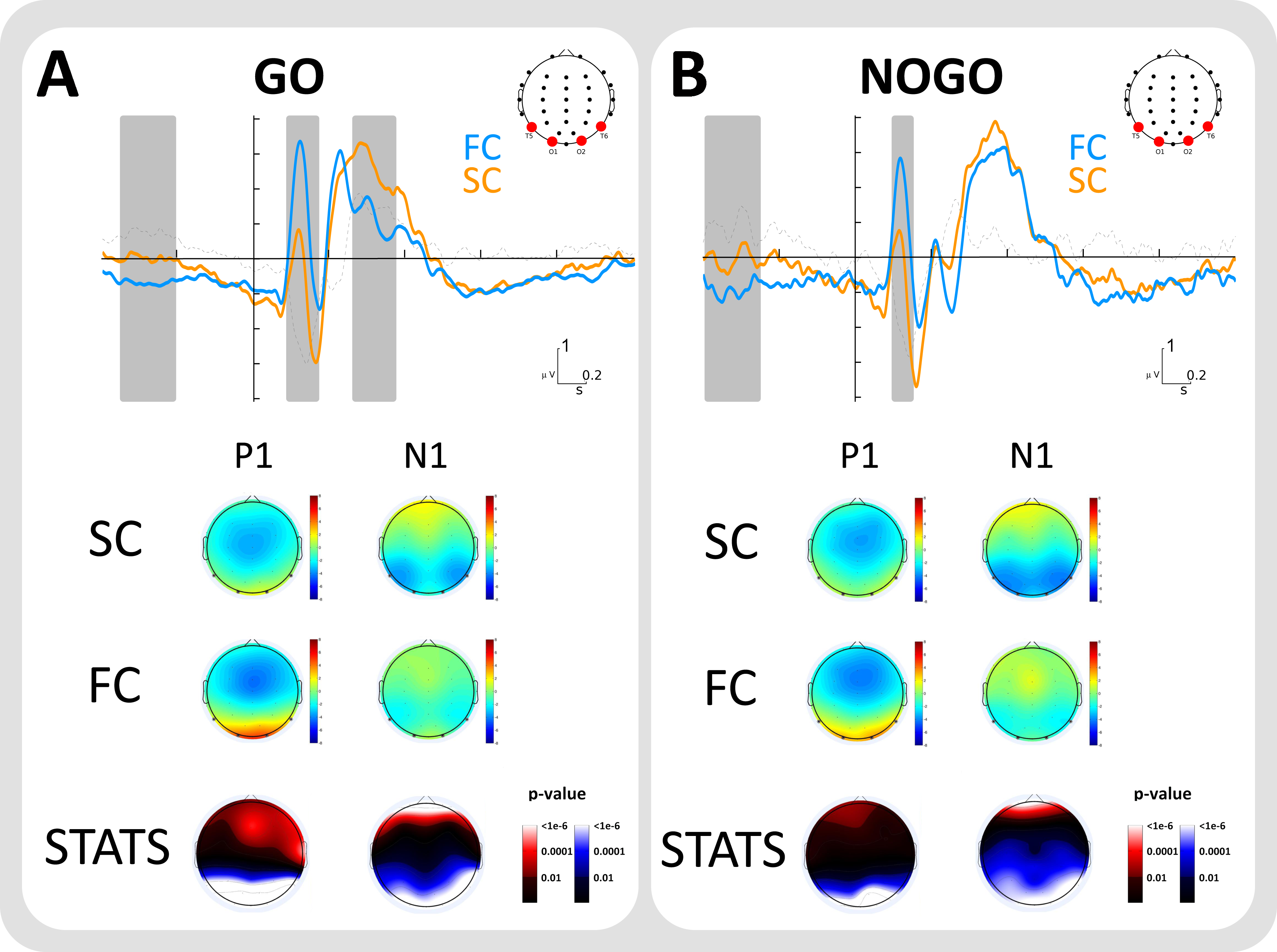

Supplement: Figure S1 — Visual ERP components. A. ERP elicited by Go trials in FC (blue line) and SC (orange line). B. ERP elicited by Nogo trials in FC (blue line) and SC (orange line). A, B. ROI is depicted in the superior right corner of each figure. The gray bar indicates regions of statistical significance (p<0.05, cluster based permutation test). The lower panel shows the P1 and N1 topographical representations for both FC and SC, and its statistical differences. (TIF) [file pone.0087232.s001.tif]
